# Pathway-dependent cold activation of heat-responsive TRPV channels

**DOI:** 10.1038/s41598-025-29524-y

**Published:** 2025-12-01

**Authors:** Guangyu Wang

**Affiliations:** 1 Department of Physiology and Membrane Biology, University of California School of Medicine, Davis, CA USA; 2 Department of Drug Research and Development, Institute of Biophysical Medico-Chemistry, Reno, NV USA

**Keywords:** Biochemistry, Biophysics, Biotechnology, Chemical biology, Computational biology and bioinformatics, Neuroscience, Physiology, Structural biology, Chemistry, Mathematics and computing

## Abstract

The homotetrameric thermosensitive transient receptor potential vanilloid 1–4 (TRPV1-4) channels in sensory neurons are highly responsive to environmental heat stimuli. However, their primary heat sensors or triggers for heat activation have not been examined for cold activation. In this computational study, cold activation of minimal TRPV1 without the pore turret was compared with that of full-length human TRPV3. The former followed a pathway from the putative heat activation starter, while the latter tracked a different pathway starting far from the assumed heat activation point. The results showed that disrupting the highly conserved intersubunit interactions near the lower gate was necessary for final channel opening. Further, the former with the same starter shared temperature sensitivity with heat activation while the latter with the different triggers did not. Therefore, this mirrored thermosensitivity, together with the matched thresholds, can be used to confirm the location of the primary thermal sensor for TRPV1 or TRPV3, and further to define the primary thermal sensor of thermosensitive TRPV4 or TRPV2 once the same heat capacity mechanism is applied.

**Supplementary Information:**

The online version contains supplementary material available at 10.1038/s41598-025-29524-y.

## Introduction

The polymodal thermosensitive transient receptor potential (TRP) channels, such as TRP vanilloid 1-4 (TRPV1-4), TRP melastatin 2-5, 8 (TRPM2-5/8), TRP ankyrin 1 (TRPA1) and TRP canonical 5 (TRPC5), function as biological thermometers to detect changes in environmental temperature. They exhibit a high temperature coefficient or sensitivity (Q_10,_ the rate of activity increase of TRP channels over a 10 °C change) and have specific activation thresholds within a temperature range of 17 °C to 53 °C^1–16^. While various regions have been suggested as the primary thermal sensors or triggers for specific thresholds, including the ankyrin repeat domain (ARD), pre-S1 domain, S1-S4 domain or voltage sensor-like domain (VSLD), pore domain, TRP helix and C terminal domain (CTD)^17–38^, recent temperature-dependent cryogenic electron microscopy (cryo-EM) structures of native TRPV1, TRPV3 and TRPM8 channels have revealed a global cooperative conformational change across all these regions during channel opening^39–42^. Furthermore, recent data from differential scanning calorimetry (DSC) and electrophysiological recordings have indicated the same specific temperature thresholds for both channel opening and melting in either TRPV1 or TRPV2^43,44^. Therefore, the heat-induced unfolding of a particular noncovalent interaction is essential for initiating channel opening.

Recently, a graph theory-based highly-sensitive grid thermodynamic model has been developed to identify the weakest noncovalent interaction responsible for the melting threshold of a protein^45–54^. In this model, tertiary noncovalent interactions identified in the high-resolution 3D structure of the protein can form an adjacency matrix or a systematic fluidic grid-like mesh network along the single polypeptide chain. In this network, each node represents a protein residue and two nodes are linked as an edge if a pair of protein residues form a noncovalent interaction via their side chains. For each noncovalent interaction, the shortest round path between two nodes along the single polypeptide chain and other edges can generate a topological grid constrained to regulate the melting temperature threshold (T_m,th_) of that interaction. Thus, when the constrained grid with the path length as its size is described as a thermo-sensitive ring or a thermo-ring with the minimum energy needed to stabilize the weakest interaction within it, the T_m,th_ for the heat unfolding of a given protein can be calculated by the grid size and the strength of the least-stable noncovalent interaction typically found in the biggest grid. In addition, the systematic thermal instability (T_i_) or flexibility can be computed from the ratio of the total unshared grid sizes (*S*) to the total tertiary noncovalent interactions (*N*) along the same single polypeptide chain. Finally, the structural temperature sensitivity (Ω_10_) can be defined as a change in the total chemical potentials of grids upon a change in the total noncovalent interactions along the same gating pathway. Since three parameters are consistent with the experimental values, the weakest noncovalent link along the lipid-dependent minimal gating pathway may serve as the primary thermal sensor or starter for thermo-gated channel activation of TRPV1, TRPV3 or TRPM8^48–50^. However, TRP channels are homo-tetramers and the inter-subunit interaction may also act as a primary thermal sensor or starter to initiate channel activation. Given that the heat and cold unfolding of any protein from the same starter has the same or comparable temperature coefficient^55^, this principle can be used to examine the primary thermal sensor or starter of thermosensitive TRPV1-4 channels.

In this study, the intra-subunit active vanilloid site of the minimal thermosensitive rat TRPV1 (rTRPV1) channel without the unstructured pore turret (604-626) was utilized as a positive control. The aim was to determine if removing phosphatidylinositol (PI) from this site disrupts the nearby weakest noncovalent interaction along the PI-dependent minimal gating pathway from the pre-S1 domain to the TRP domain, initiating both heat and cold activation with a shared temperature coefficient^56,57^. On the other hand, the highly conserved cytoplasmic ATP site shared among TRPV1, TRPV2 and TRPV3 was used as a negative control. The goal was to ascertain whether disrupting the highly conserved inter-subunit K169-E751’ salt bridge at the interface between the ARD and the CTD’ in reduced human TRPV3 (hTRPV3) through the K169A mutation triggers cold activation, which has an unshared temperature coefficient compared to the heat activation, which is induced by breaking the weakest noncovalent interaction in the pore domain along the phosphatidylcholine (PC)-dependent minimal gating pathway from the pre-S1 domain to the TRP domain^58–60^. Meanwhile, this study also investigated how each pathway could ultimately lead to channel opening or activation from each primary point of initiation.

The results demonstrated that the primary thermal sensor or trigger actually originates from the weakest intra-subunit noncovalent interaction within the single polypeptide chain rather than any other inter-subunit noncovalent interactions. However, disrupting the highly conserved intersubunit interactions between an aromatic amino acid residue (Tyr/His) in the S4-S5 linker and a positively charged residue (Arg/Lys) on S5 near the lower gate was required for the final channel opening. These findings were then employed to identify the potential primary thermal sensor in human TRPV4 or TRPV2 (hTRPV4 or hTRPV2) once the calculated T_m,th_ and Ω_10_ matched the experimental threshold and reflected thermosensitivity Q_10_. The extensive implications of thermo-gated activation pathways of TRPV1-4 biothermometers were also discussed.

## Results

### Disrupting the highly conserved intersubunit bridges near the lower gate is required for the final heat activation of TRPV1 and TRPV3

Recent studies have identified the weakest tertiary noncovalent interactions along the PI/PC-dependent minimal gating pathways as the primary thermal starters with matched thresholds for initiating heat activation of TRPV1 or TRPV3^48,49^. However, it is unclear how the channel is finally activated by heat. Given that a weak acid HoAC can protonate H521 in the S4-S5 linker and thus disrupt its swapping π interaction with R539’ on S5 near the lower gate for channel activation of rat TRPV2 (rTRPV2) but a corresponding disulfide bond between Y602C and R616C locks hTRPV4 in a closed state^41,61,62^, it is of interest to examine if disrupting the highly conserved intersubunit bridges near the lower gate is necessary for the final heat activation of TRPV1 and TRPV3.

**Fig. 1:**
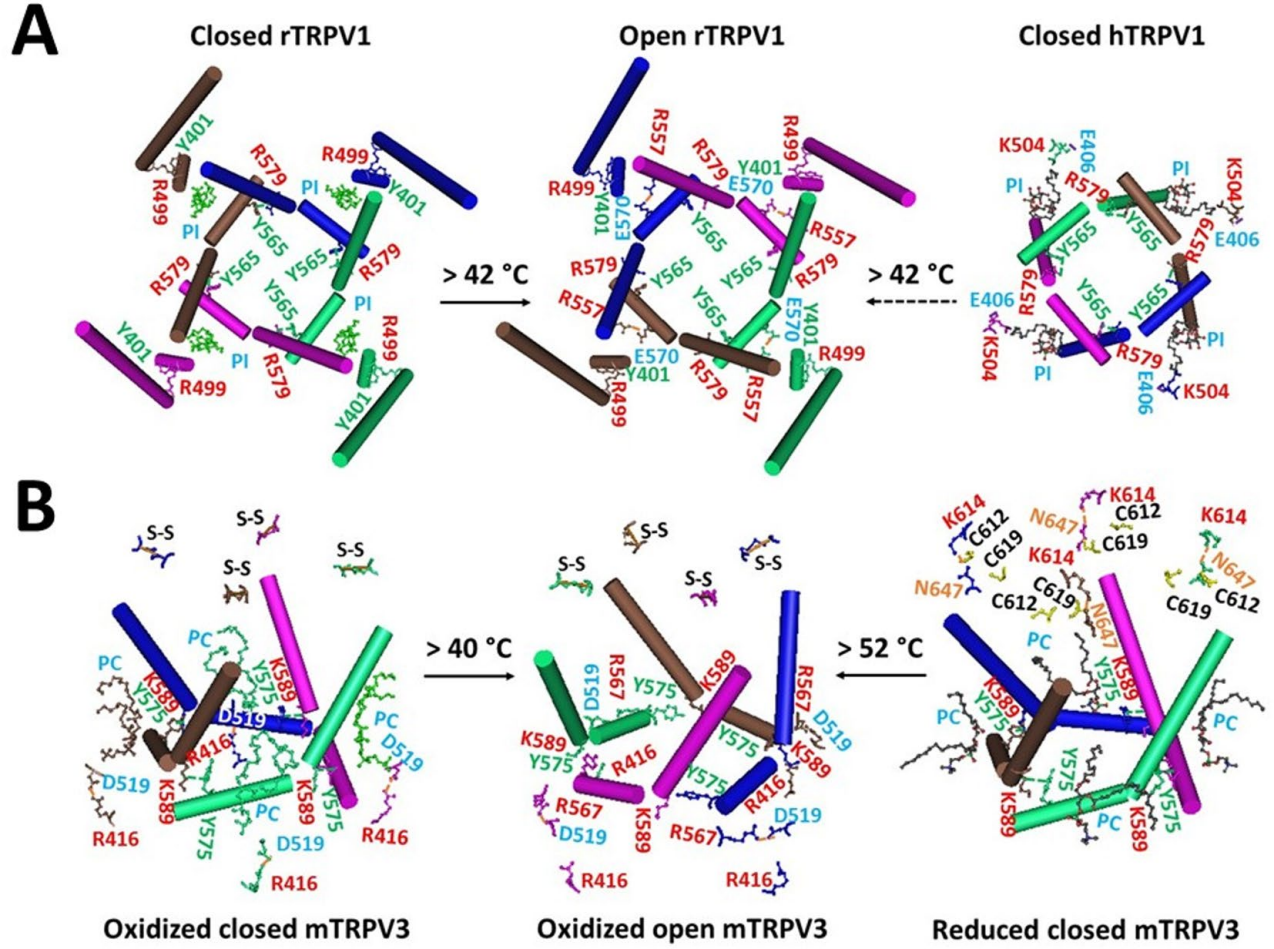
Allosteric gating coupling between the weakest intrasubunit bridges and the highly conserved intersubunit bridges near the lower gates of TRPV1 (**A**) and TRPV3 (**B**). The cryo-EM structures of closed (PDB: 7LPC) and open (PDB: 7LPE) rTRPV1 in MSP2N2 at 48 °C and closed (PDB: 7MIN) and open (PDB: 7MIO) mTRPV3 with the C612-C619 disulfide bond in cNW11 at 42 °C were utilized for the model. Closed and reduced hTRPV1 in cNW11 (PDB: 8GF9) and mTRPV3 (PDB: 6LGP) in MSP2N2 at 4 °C were used as controls. Disrupting the weakest Y401-R499/K504-E406 bridges in TRPV1 or the weakest R416-D519/K614-N647 bridges in TRPV3 disconnected the Y565-R579’ swapping bridges in TRPV1 or the Y575-K589’ swapping bridges in TRPV3 during heat activation, respectively.

The primary investigation revealed that in the full-length closed rTRPV1 channel at 48 °C, the swapping K155-E761’ salt bridges were disrupted (PDB: 7LPC)^40^. However, along with the weakest Y401-R499 π interactions at the pre-S1/VSLD interface and PI at the vanilloid sites, Y565 in the S4-S5 linkers from one subunit still formed swapping π interactions with nearby R579’ on S5 from the other subunit (Fig. 1A). Similarly, in closed hTRPV1 (PDB: 8GF9), the weakest K504-E406 salt bridge at the pre-S1/VSLD interface and PI at the vanilloid sites also coexisted with the swapping Y565-R579’ bridge. When the weakest Y401-R499 or K504-E406 bridges were unfolded along with PI release from the vanilloid site in the heat-evoked open state above the threshold of 42 °C, the swapping Y565-R579’ bridges were also disrupted (PDB: 7LPE) (Fig. 1A).

Furthermore, the corresponding swapping Y575-K589’ π interactions and PC at the vanilloid sites were also accompanied by the weakest K614-N647 bridges in reduced and closed mTRPV3 (PDB: 6LGP) or the least-stable R416-D519 bridges in oxidized and closed mTRPV3 (PDB: 7MIN). However, they all disconnected along with the PC released from the vanilloid sites in the heat-evoked open state (PDB: 7MIO) (Fig. 1B).

Therefore, regardless of where the activation pathway begins in response to various physical or chemical stimuli, disrupting the highly conserved intersubunit bridges near the lower gate is essential for the final heat activation of TRPV1 or TRPV3.

### Reduced closed rTRPV1-Δ(604-826) in MSP2N2 has a corresponding melting threshold to release PI from the vanilloid site for heat activation

**Fig. 2:**
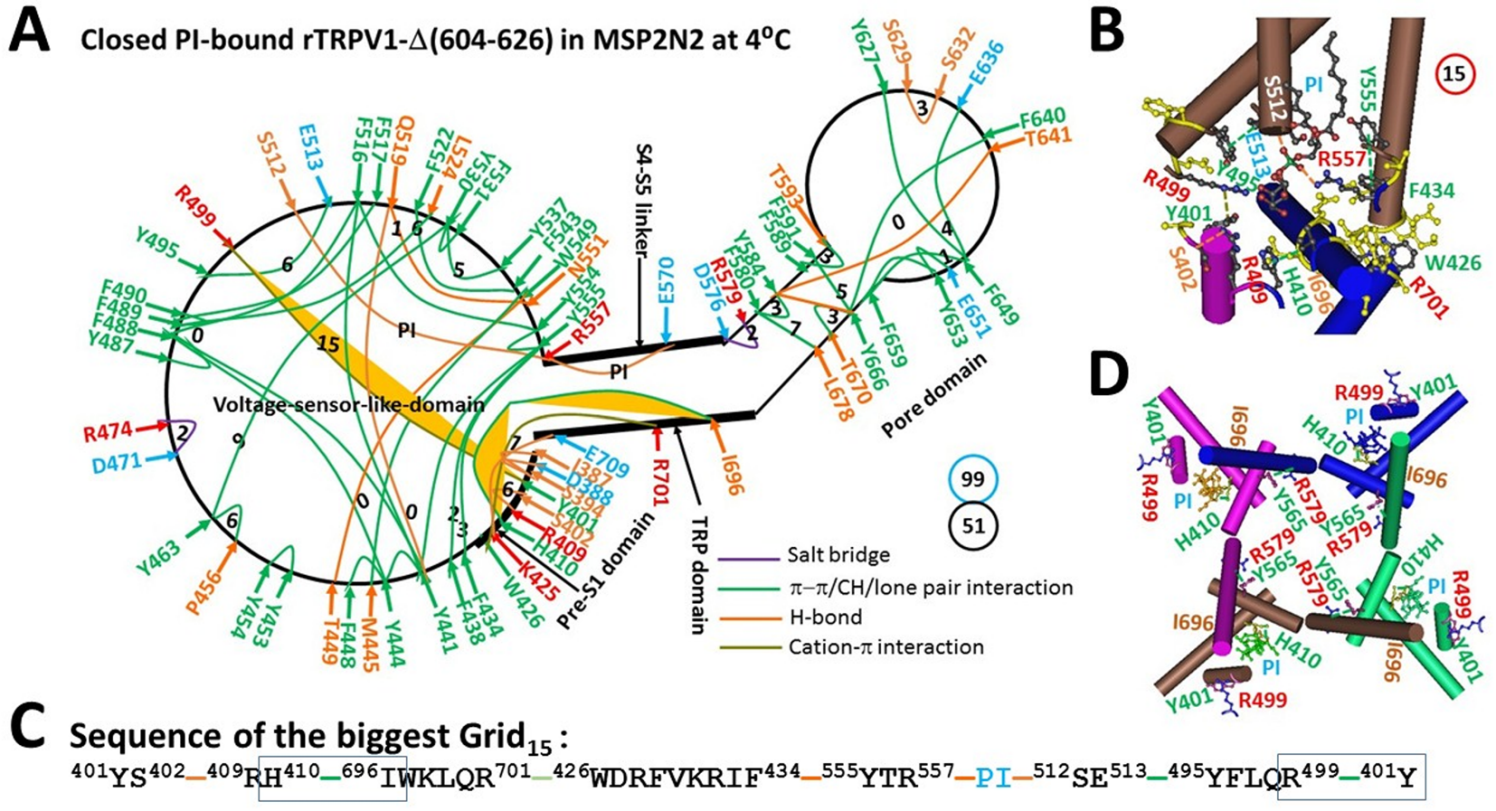
Thermoring structures of closed rTRPV1-Δ(604-626) at low temperature. (**A**) The grid-like noncovalently interacting mesh network along the PI-dependent minimal gating pathway of a single subunit of reduced and closed rTRPV1-Δ(604-626) in MSP2N2 at 4 °C (PDB: 5IRZ). The pore domain, the S4-S5 linker, the TRP domain, and the pre-S1 domain are indicated by black arrows, except the VSLD. Salt bridges, π interactions, and H-bonds between paired amino acid side chains along the PI-dependent minimal gating pathway from I387 to K710 are denoted in purple, green, and orange, respectively. The specific constrained grid sizes necessary to regulate the least-stable noncovalent interactions in the grids are indicated with black numbers. The identified weakest Y401-R499 and H410-I696 bridges in the biggest Grid_15_ are emphasized in orange. The total grid sizes and the total grid size-controlled noncovalent interactions along the PI-dependent minimal gating pathway are displayed in cyan and black circles, respectively. (**B**) The structure of the identified biggest Grid_15_ with a 15-residue size to regulate the weakest Y401-R499 and H410-I696 bridges at the TRP/pre-S1/VSLD interfaces. (**C**) The sequence of the biggest Grid_15_ to control the weakest Y401-R499 and H410-I696 bridges highlighted in the blue boxes. (**D**) Allosteric gating coupling between the weakest intrasubunit Y401-R499/H410-I696 bridges and the Y565-R579’ swapping interactions near the lower gate in the closed state.

When the pore turret (604–626) is removed from rTRPV1, this minimal thermosensitive channel is structured from N-terminal T335 to C-terminal T751 in membrane scaffold protein 2N2 (MSP2N2) at 4 °C (PDB: 5IRZ)^56^. When compared with the full-length closed rTRPV1 channel in MSP2N2 at 48 °C (PDB: 7LPC), most of the noncovalent interactions were conserved except for small fractional changes along the PI-dependent minimal gating pathway from I387 in the pre-S1 domain to K710 in the TRP domain (Fig. 2A). Notably, when the D576-T685 H-bond in the pore domain was disconnected, the R409-D509 salt bridge and the W426-F434 π interaction at the interface between the VSLD and the pre-S1 domain were also broken, along with the PI binding to S512, R557 and E570 at the interface between the S4-S5 linker and the VSLD^48^. Therefore, when the total noncovalent interactions and grid sizes were 51 and 99, respectively (Fig. 2A, Table S1), the systematic thermal instability (T_i_) was about 1.94 (Table 1).

**Table 1:** Comparison of local cold-induced thermoring structural changes of *apo* TRPV1 or TRPV3 in MSP2N2 along the PI/PC-dependent minimal gating pathway from D388 to K710 or from D396 to K705.

PDB ID	5IRZ	8U3L	6UW4	6UW6
Construct	rTRPV1-Δ(604-626)	rTRPV1-Δ(604-626)	hTRPV3	hTRPV3-K169A
Lipid at the vanilloid site	bound	free	present	free
Sampling temperature, °C	4	25	4	4
Gating state	Closed	Open	Closed	Open
# of the biggest Grid_s_	Grid_15_	Grid_9_	Grid_14_	Grid_23_
grid size (s)	15	9	14	23
# of energetically equivalent basic H-bonds (n) controlled by Grid_s_	2.0	0.5	2.5	1.5
Total non-covalent interactions (*N*)	51	43	52	38
Total grid sizes (*S*), a.a	99	73	98	97
Systemic thermal instability (T_i_)	1.94	1.70	1.88	2.55
Calculated T_m,th_, °C	**44**	**41**	**51**	**23**
Experimental T_m,th_, °C	**43**	** < 41**	**50**	** < 25**
Calculated Ω_10,cold_ at E = 1 kcal/mol		21.2		0.39
Experimental Q_10,heat_		21.8		22.2
Refs for Experimental T_m,th_, °C and Q_10_	^63^	^63^	^32^	^60,75^

The comparative parameters are highlighted in bold.

On the other hand, the biggest Grid_15_, rather than the biggest Grid_13_, was found to control not only the least-stable Y401-R499 cation-π interaction at the pre-S1/VSLD interface but also the least-stable H410-I696 π interaction at the pre-S1/TRP interface through a different thermoring pathway. It cycled through Y401 to S402, R409, H410, I696, R701, W426, F434, Y555, R557, PI, S512, E513, Y495, R499, and back to Y401 (Fig. 2B–C). Therefore, the weakest Y401-R499 and H410-I696 bridges were closely related to PI binding. When these two bridges were energetically equivalent to 2.0 basic H-bonds (2.0 kcal/mol), the calculated melting temperature threshold (T_m,th_) was approximately 44 °C, matching the experimental value of 43 °C for heat activation^63^. Meanwhile, the swapping Y565-R579’ π interactions were also present near the lower gate (Fig. 2D). Accordingly, the heat-induced unfolding of the weakest Y401-R499 and H410-I696 bridges was essential to release PI from the active vanilloid site and then to disrupt the swapping Y565-R579’ bridges, allowing channel opening above 43 °C (Fig. 2A)^40,48^. The next question is whether the removal of PI also disrupts the same weakest Y401-R499 and H410-I696 bridges to faciliate channel opening at lower temperatures with a shared thermosensitivity.

### Removal of PI from reduced rTRPV1-Δ(604-826) in MSP2N2 induces cold activation with a shared thermosensitivity

**Fig. 3:**
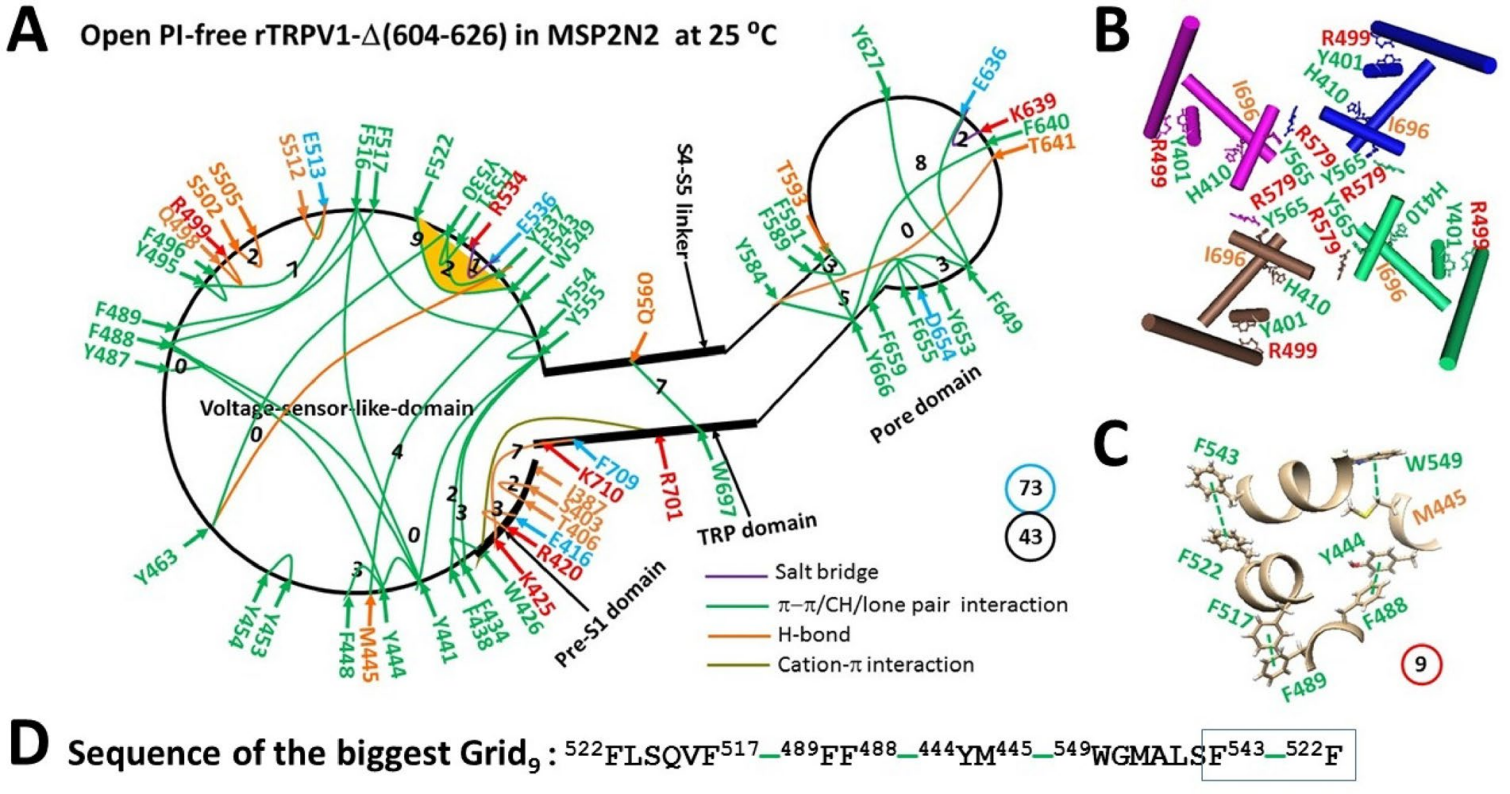
Thermoring structural rearrangement of rTRPV1-Δ(604-626) upon PI release at low temperature. (**A**) The grid-like noncovalently interacting mesh network along the PI-dependent minimal gating pathway of a single subunit of reduced and open rTRPV1-Δ(604-626) in MSP2N2 at 25 °C (PDB: 8U3L). The pore domain, the S4-S5 linker, the TRP domain, and the pre-S1 domain are indicated by black arrows, except the VSLD. Salt bridges, π interactions, and H-bonds between paired amino acid side chains along the PI-dependent minimal gating pathway from I387 to K710 are denoted in purple, green, and orange, respectively. The specific constrained grid sizes necessary to regulate the least-stable noncovalent interactions in the grids are indicated with black numbers. The identified weakest F522-F543 π-π interaction in the biggest Grid_9_ is emphasized in yellow. The total grid sizes and grid size-controlled noncovalent interactions along the PI-dependent minimal gating pathway are displayed in cyan and black circles, respectively. (**B**) Disrupting the weakest Y401-R499 and H410-I696 bridges diconnected the Y565-R579’ swapping interactions near the lower gate for channel opening. (**C**) The structure of the biggest Grid_9_ with a 9-residue size to regulate the weakest F522-F543 π-π interaction in the VSLD. (**D**) The sequence of the biggest Grid_9_ to control the weakest F522-F543 π-π interaction highlighted in the blue box.

When the PI lipid was removed from the active vanilloid site to disrupt the weakest Y401-R499 and H410-I696 bridges, as well as the S512-PI-R557-PI-E570 bridges along the PI-dependent minimal gating pathway from I387 to K710, rTRPV1-Δ(604-826) opened in MSP2N2 at 25 °C with a global cooperative conformational change (Fig. 3A). Overall, the totals of noncovalent interactions and grid sizes decreased from 51 and 99 to 43 and 73, respectively (Figs. 2A & 3A, Table S1 & S2). Consequently, the systematic thermal instability (T_i_) decreased from 1.94 to 1.70 (Table 1). Of particular interest, the calculated cold thermosensitivity (Ω_10,cold_) was 21.2, which was comparable to the measured heat thermosensitivity (Q_10,heat_) of 21.8 (Table 1)^63^.

On the other hand, in addition to the swapping Y565-R579’ bridge being broken near the lower gate (Fig. 3B), the new biggest Grid_9_ was introduced to control the least-stable F522-F543 π interaction in the VSLD through a thermoring from F522 to F517, F489, F488, Y444, M445, W549, F543, and back to F522 (Fig. 3C–D). This bridge was highly conserved in gating states of rTRPV1, regardless of the deletion of the pore turret from 604 to 626^48^. Therefore, it may play an important role in stabilizing the rTRPV1 channel. When this weakest bridge energetically equated to 0.5 basic H-bond (0.5 kcal/mol), the calculated melting threshold (T_m,th_) was approximately 41 °C (Table 1). Since no grid size exceeded 9 between the C- and N-termini beyond the PI-dependent minimal gating pathway from D388 to K710 (Figure S1), this open state induced by PI removal at low temperatures may only occur below 41 °C.

### Reduced closed hTRPV3 in MSP2N2 has a matching melting threshold for the initial heat activation

**Fig. 4:**
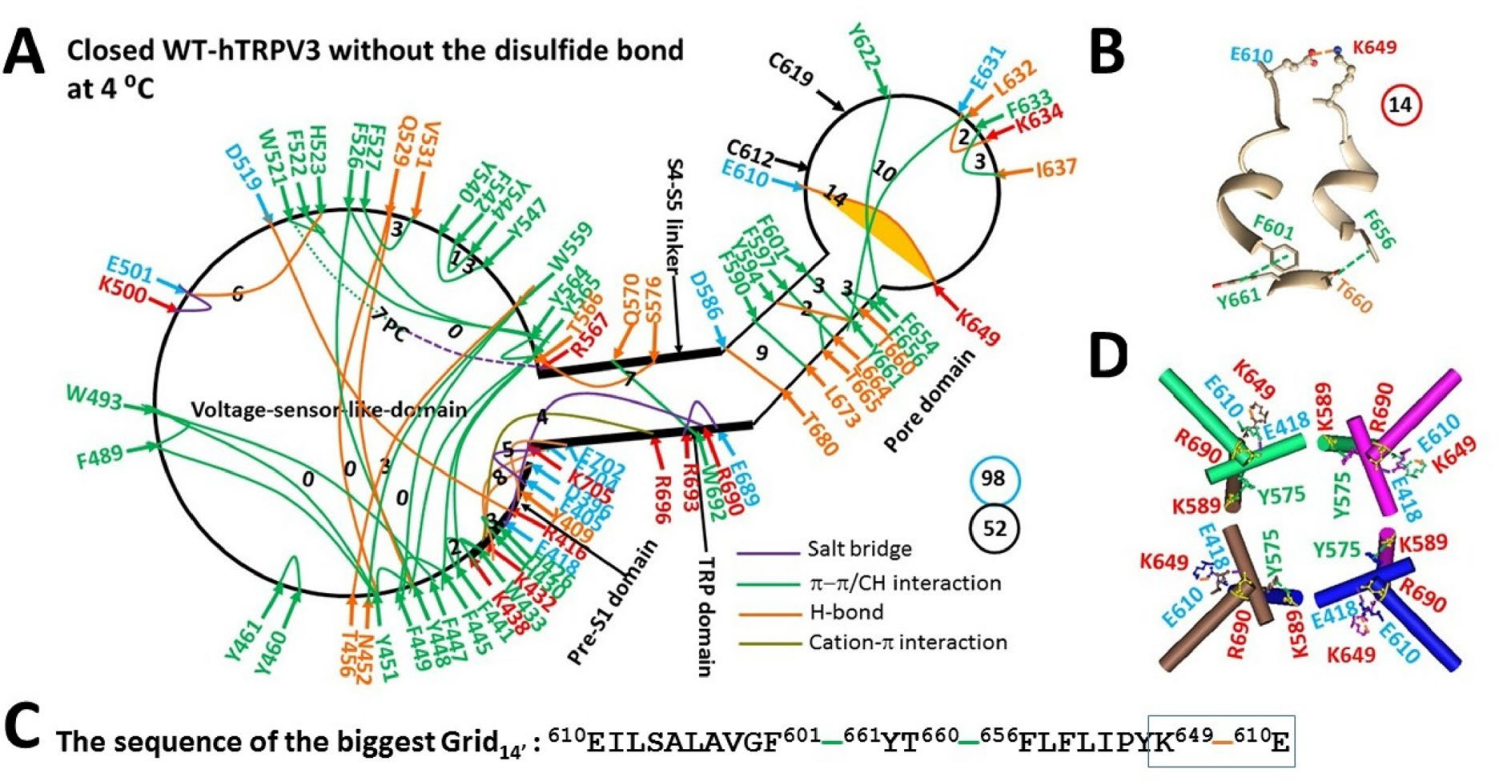
Thermoring structures of closed hTRPV3 at low temperature. (**A**) The grid-like noncovalently interacting mesh network along the PC-dependent minimal gating pathway of a single subunit of reduced and closed hTRPV3 in MSP2N2 at 4 °C (PDB: 6UW4). The pore domain, the S4-S5 linker, the TRP domain, and the pre-S1 domain are indicated by black arrows, except the VSLD. Salt bridges, π interactions, and H-bonds between paired amino acid side chains along the PC-dependent minimal gating pathway from D396 to K705 are denoted in purple, green, and orange, respectively. The specific constrained grid sizes necessary to regulate the least-stable noncovalent interactions in the grids are indicated with black numbers. The identified weakest E610-K649 H-bond in the biggest Grid_14_ is emphasized in orange. The dashed line represents the putative PC bridge between W521 and R567. The total grid sizes and the total grid size-controlled noncovalent interactions along the PC-dependent minimal gating pathway are displayed in cyan and black circles, respectively. (**B**) The structure of the biggest Grid_14_ with a 14-residue size to regulate the wesakest E610-K649 H-bond in the pore domain. (**C**) The sequence of the biggest Grid_14_ to control the weakest E610-K649 H-bond highlighted in the blue box. (**D**) Allosteric gating coupling between the weakest intrasubunit E610-K649 bridges and the Y575-K589’ swapping interactions near the lower gate in the closed state, along with the E418-R690 salt bridges at the protein-water interface.

Reduced hTRPV3 exhibited structural similarities to reduced mTRPV3 at 4 °C, including the active vanilloid site for the same putative phosphatidylcholine (PC) lipid binding through a π interaction with W521 and a salt bridge with R567 (Fig. 4A)^39,49,60^. However, some differences were observed throughout the entire polypeptide chain. For example, when the D519-R698 salt bridge at the VSLD/TRP interface was replaced with the E418-R690 salt bridge at the pre-S1/TRP interface, the H-bond moved from T397-E704 to Y409-E702 together with the formation of the E405-K705 salt bridge. Meanwhile, the Q570-W692 π interaction was present at the S4-S5 linker/TRP interface. As a result, the total noncovalent interactions and the total grid sizes along the PC-dependent minimal gating pathway from D396 in the pre-S1 domain to K705 in the TRP domain increased from 51 to 52 and from 96 to 98, respectively (Fig. 3A, Table S3). In this case, the systematic thermal instability (T_i_) remained at 1.88 (Table 1)^49^.

Notably, the R416-D519 H-bond at the pre-S1/VSLD interface was not controlled by the smaller thermoring (Grid_4_) from R416 to T411, D519 and back to R416^49^. Instead, it was governed by the smaller thermoring (Grid_3_) from R416 to E418, R690, E689, R693, W692, Q570, R567, PC, W521, D519 and back to R416 (Fig. 4A). Since no grid size was bigger than 14 between the C- and N-termini beyond the PC-dependent minimal gating pathway from D396 to K705 (Figure S1), Grid_14_ in the pore domain was still the biggest (Fig. 4A). It had the shortest circular path from E610 to F601, Y661, T660, F656, K649, and back to E610 to control the least-stable E610-K649 H-bond/salt bridge between two pore turrets (Fig. 4B–C). With 2.5 equivalent basic H-bonds being energetically equivalent to this weakest bridge, the calculated T_m,th_ for heat unfolding was at least 51 °C (Table 1), which was close to the measured threshold of 50 °C for the heat-induced channel opening of the reduced hTRPV3 channel^32^. Meanwhile, the swapping Y575-K589’ π interactions appeared near the lower gate (Fig. 4D).

### Disrupting the inter-subunit K169-E751’ salt bridge in reduced hTRPV3 in MSP2N2 induces cold activation with unique thermosensitivity

If heat-evoked hTRPV3 activation begins with the disruption of the intersubunit K169-E751ʼ bridge rather than the weakest intra-subunit E610-K649 bridge, then the same thermosensitivity for heat activation should be observed for cold activation. To test this hypothesis, the thermoring structures of the K169A-induced open state at 4 °C were analyzed.

**Fig. 5:**
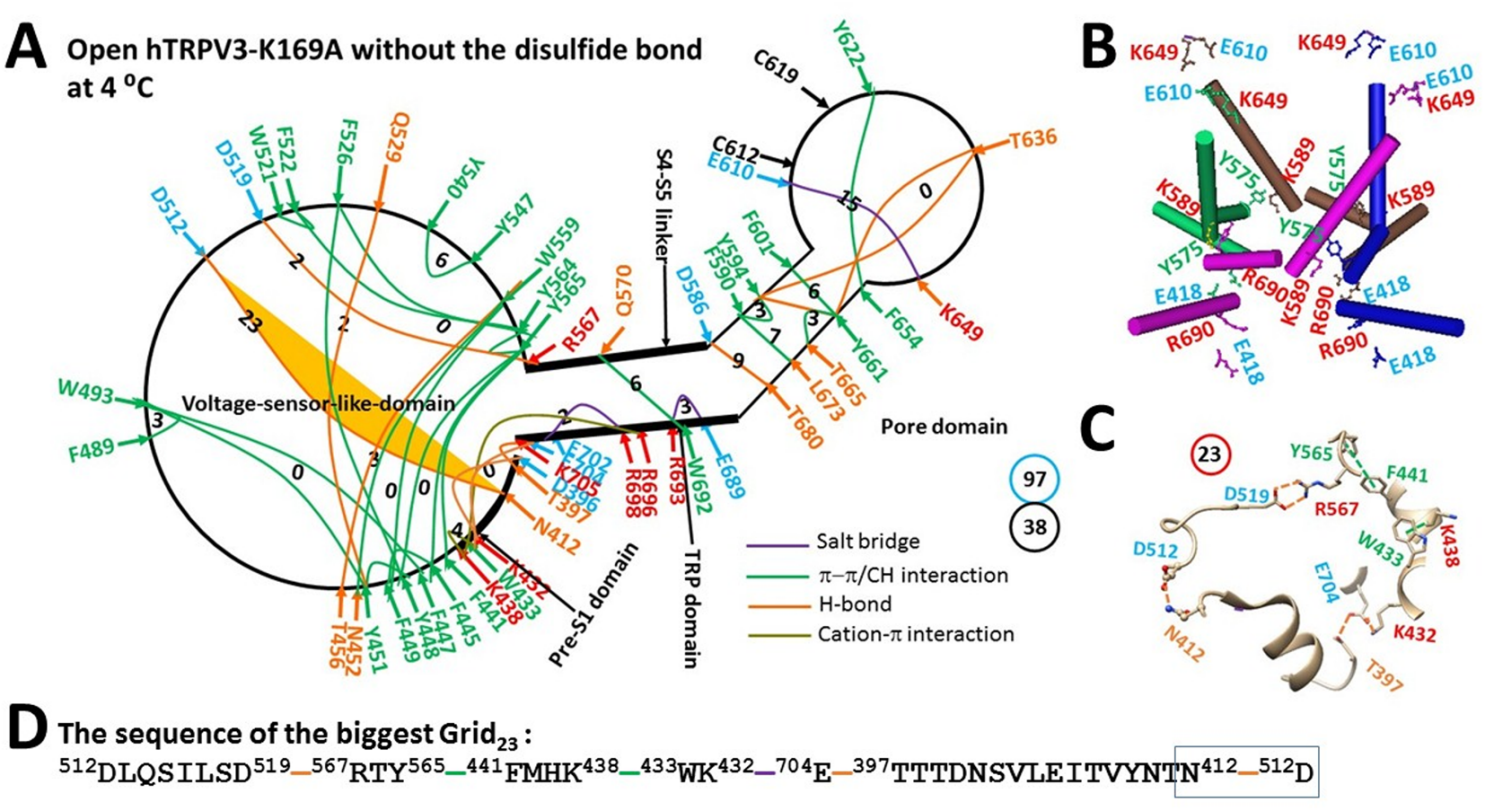
Thermoring structural rearrangement of hTRPV3 upon the K169A mutation at low temperature. (**A**) The grid-like noncovalently interacting mesh network along the PC-dependent minimal gating pathway of of a single subunit of reduced and open hTRPV3-K169A in MSP2N2 at 4 °C (PDB: 6UW6). The pore domain, the S4-S5 linker, the TRP domain, and the pre-S1 domain are indicated by black arrows except the VSLD. Salt bridges, π interactions, and H-bonds between paired amino acid side chains along the PC-dependent minimal gating pathway from D396 to K705 are marked in purple, green, and orange, respectively. The specific constrained grid sizes needed to control the least-stable noncovalent interactions in the grids are labeled with black numbers. The identified weakest N412-D512 H-bond in the biggest Grid_23_ is highlighted. The total grid sizes and the total grid size-controlled noncovalent interactions along the PC-dependent minimal gating pathway are shown in the cyan and black circles, respectively. (**B**) Disrupting the E418-R690 bridges disconnected the Y575-K589’ swapping interactions near the lower gate rather than the weakest E610-K649 bridges for channel opening by cold. (**C**) The structure of the biggest Grid_23_ with a 23-residue size to control the weakest N412-D512 H-bond at the pre-S1/VSLD interface. (**D**) The sequence of the biggest Grid_23_ to control the weakest N412-D512 H-bond highlighted in the blue box.

When the K169A mutation disrupted the highly-conserved K169-E751ʼ salt bridge swap at the ATP site^58–60^, the R416-D519 H-bond at the pre-S1/VSLD interface, as well as the E418-R690 salt bridge at the pre-S1/TRP interface, were also broken at 4 °C (Fig. 5A). Consequently, despite the intact E610-K649 salt bridge found in the biggest Grid_14_ of the closed state (Fig. 4A–B), the swapping Y575-K589’ bridges were disrupted for channel opening (Fig. 5B), along with the new biggest Grid_23_ at the VSLD/pre-S1/TRP interfaces due to the absence of a grid size bigger than 23 between the C- and N-termini beyond the PC-dependent minimal gating pathway from D396 to K705 (Figs. 4B–C & S1). This Grid_23_ had a size of 23 free residues via the shortest circular path from D512 to D519, R567, Y565, F441, K438, W433, K432, E704, T397, N412 and back to D512 to regulate the least-stable D512-N412 H-bond at the pre-S1/VSLD interface (Fig. 4D). When this weakest H-bond was energetically equivalent to 1.5 basic H-bonds, the melting temperature threshold (T_m,th_) for heat unfolding was estimated to be around 23 °C (Table 1). Therefore, the K169A mutant could still be open up to 23 °C.

On the other hand, after the release of the putative PC lipid from the active vanilloid site, the stimulatory D519-R567 H-bond was formed along with a decrease in the total noncovalent interactions and total grid sizes along the PC-dependent minimal gating pathway from D396 to K705 from 52 and 98 to 38 and 97, respectively (Figs. 4A & 5A, Tables S3 & S4)^49^. This led to an increase in systematic thermal instability (T_i_) from 1.88 to 2.55 (Table 1). In this regard, the K169A-induced open state was actually unstable even at 4 °C. Notably, the calculated cold sensitivity (Ω_10,cold_) was about 0.39, significantly lower than the experimental mean heat sensitivity (Q_10,heat_) of 22.2 (Table 1)^32^. Therefore, the heat activation of reduced TRPV3 was initiated from the unfolding of not the inter-subunit K169-E751ʼ salt bridge but the weakest E610-K649 H-bond in the pore domain (Fig. 4).

Taken together, when the weakest tertiary noncovalent interactions in thermosensitive TRPV1 and TRPV3 were identified as primary thermal triggers with thresholds that matched theoretically and experimentally, their unfolding at low or high temperatures, together with the swapping Y565-R579’or Y575-K589’ π interactions near the lower gate, could finally activate the channels with mirrored cold and heat sensitivities according to the heat capacity mechanism^55^.

TRPV1-4 channels share the same topology: six transmembrane segments (S1-S6), a pore-loop region between S5 and S6, a S4-S5 linker, the TRP domain and intracellular N- and C-terminal tails^39–41,56^. Given that the same heat capacity mechanism applies to TRPV1-4 channels with some sequence homology, matched thresholds and thermosensitivties shared by both cold and heat activations can be used to define or confirm primary thermal sensors in hTRPV1-4 channels. For hTRPV1, the weakest E406-K504 (E405-K504 in rTRPV1) bridge at the internal pre-S1/VSLD interface also has a matched T_m,th_ of 41 °C (Fig. 1A)^48,64^. If the same open state is shared between hTRPV1 and rTRPV1, the heat activation upon the unfolding of this E406-K504 bridge also exhibits a matched thermosensitivity Q_10_ of 28^48^. Hence, the weakest E406-K504 bridge can serve as a thermal sensor. For hTRPV3 without the C612-C619 disulfide bond, the weakest E610-K649 H-bond at the external interface between two pore turrets along the PC-dependent minimal gating pathway from D396 to K705 has a matching threshold of 52 °C for initial heat activation (Table 1)^32,49^. If unfolding the weakest bridge open the same oxidized channel, the matched heat sensitivity of 21 confirms the E610-K649 H-bond in the pore domain as the thermal starter^49^. Similarly, the primary potential thermal sensors of hTRPV4 and hTRPV2 can also be identified by matching thresholds and thermosensitivity for symmetric cold and heat activations even at low temperature.

### Identification of a thermal sensor in hTRPV4

**Fig. 6:**
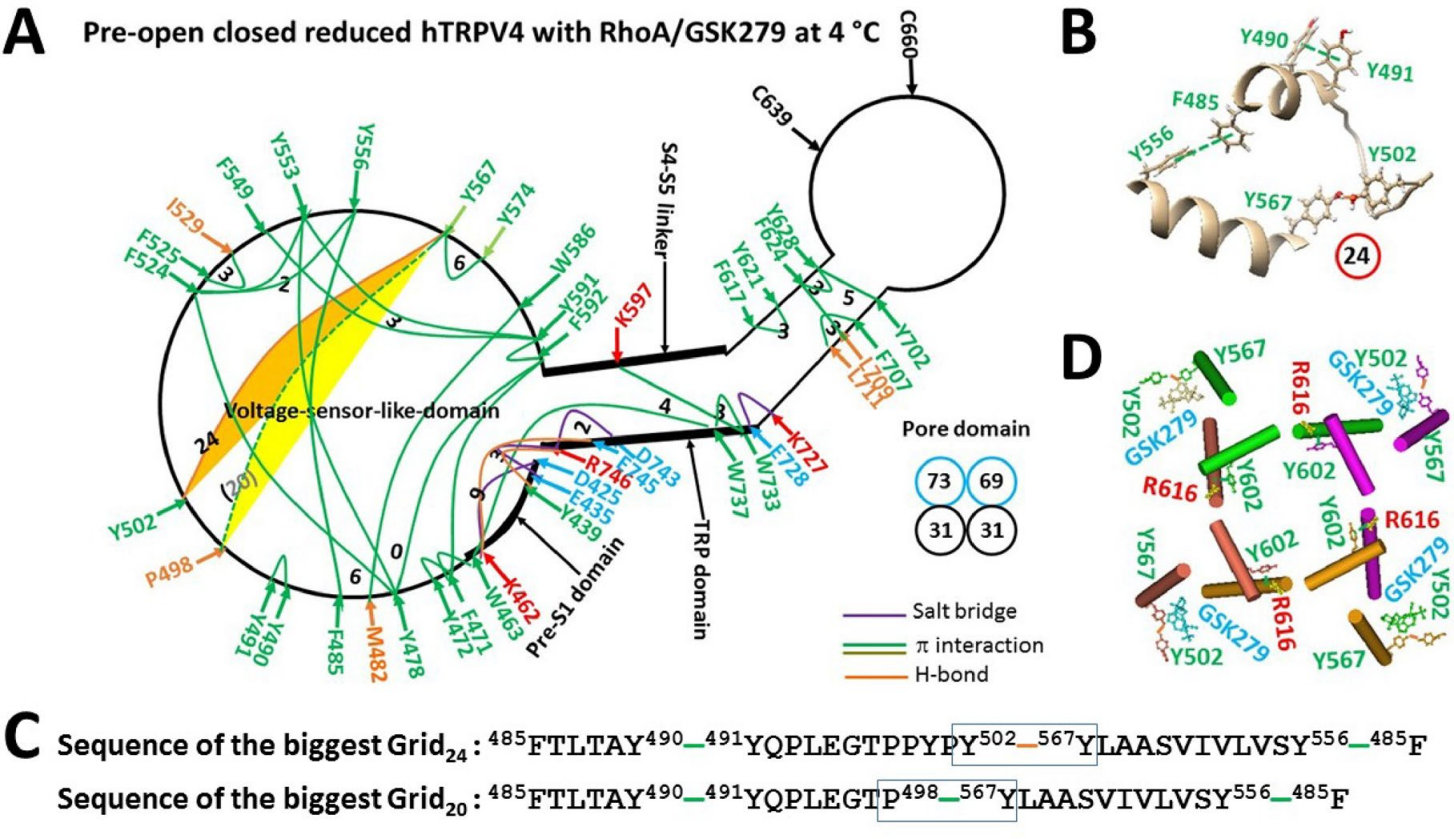
Thermoring structures of closed hTRPV4 at low temperature. (**A**) The grid-like noncovalently interacting mesh network along the minimal gating pathway of a single subunit of reduced and closed hTRPV4 with RhoA and GSK279 in MSP2N2 at 4 °C (PDB: 8FC7). The pore domain, the S4-S5 linker, the TRP domain, and the pre-S1 domain are indicated by black arrows, except the VSLD. Salt bridges, π interactions, and H-bonds between paired amino acid side chains along the minimal gating pathway from D425 to R746 are denoted in purple, green, and orange, respectively. The specific constrained grid sizes needed to regulate the least-stable noncovalent interactions in the grids are indicated with black numbers. The identified weakest Y502-Y567 H-bond in the biggest Grid_24_ is highlighted in orange. The dashed line represents the putative weakest P498-Y567 π bridge in the biggest Grid_20_. The total grid sizes and the total grid size-controlled noncovalent interactions along the minimal gating pathway are displayed in cyan and black circles, respectively. (**B**) The structure of the biggest Grid_24_ with a 24-residue size to regulate the weakest Y502-Y567 H-bond in the VSLD. (**C**) The sequence of the biggest Grid_24_ to control the weakest Y502-Y567 H-bond highlighted in the blue box. (**D**) Allosteric gating coupling between the weakest intrasubunit Y502-Y567 bridges and the Y602-R616’ swapping interactions near the lower gate in the closed state.

Given the failure of hTRPV4 heat activation in inside-out membrane patches, an intracellular inhibitory ligand is necessary for the thermal activation of hTRPV4^65^. In this case, a primary search for a thermal sensor focused on the cryo-EM structure of closed hTRPV4 with RhoA/ GSK2798745 (GSK279) bound^66^. The thermoring analyses indicated the biggest Grid_24_ in the VSLD along the minimal gating pathway from D425 to R746, controlling the weakest Y502-Y567 H-bond at the interface between S1-S2 and S3-S4 linkers via a thermoring from F485 to Y490, Y491, Y502, Y567, Y556, and back to F485 (Fig. 6A–C). When this H-bond was energetically equivalent to 2 basic H-bonds (2.0 kcal/mol), the calculated T_m,th_ was about 26 °C, similar to the experimental threshold of 24–27 °C of mouse TRPV4 (mTRPV4) as reported with HEK293 cells or *Xenopus* oocytes (Table 2)^65^. Alternatively, when the weakest P498-Y567 π interaction replaces the interfacial H-bond, the biggest Grid_24_ changed to Grid_20_ (Fig. 6A–C), and the calculated T_m,th_ to control this π interaction was about 34 °C, also close to the threshold of 34 °C of rat TRPV4 (rTRPV4) as reported in hippocampal neurons or with HEK293 cells^8,9,67^. Therefore, either Y502-Y567 or P498-Y567 bridge at the external interface between S1-S2 and S3-S4 linkers in the VSLD could serve as a primary thermal sensor with a matched threshold to detect a temperature range from 27 to 34 °C. Notably, along with the swapping Y602-R616’ bridge near the lower gate (Fig. 6D), the total noncovalent interactions and grid sizes were 31 and 73, respectively (Fig. 6A, Table S5). Therefore, the higher systematic thermal instability (T_i_) of 2.35 suggested that this closed state was unstable (Table 2).

**Table 2:** Comparison of thermo-induced thermoring structural changes of TRPV4/TRPV2 along the ligand/PE-dependent minimal gating pathway.

PDB ID	8FCA	8FC7	6U84
Construct	hTRPV4	hTRPV4/ RhoA/GSK279	hTRPV2
Lipid at the vanilloid site	free	free	free
Ligand	4a-PDD	GSK279	free
Lipid environment	LMNG/CHS	MSP2N2	
Sampling temperature, °C	4	4	4
Gating state	Open	Closed	Closed
# of the biggest Grid_s_	Grid_16_	Grid_24_	Grid_9_
grid size (s)	16	24	9
# of energetically equivalent basic H-bonds (n) controlled by Grid_s_	3.75	2.0	2.0
Total non-covalent interactions (*N*)	20	31	55
Total grid sizes (*S*), a.a	55	73	69
Systemic thermal instability (T_i_)	2.75	2.35	1.25
Calculated T_m,th_, °C	**59.5**	**26**	**61**
Experimental T_m,th_, °C	** > 43**	**24–27**	** > 60**
Calculated Ω_10,cold_ at E = 1 kcal/mol	**20**		
Experimental Q_10,heat_	**19.1**		
Refs for Experimental T_m,th_, °C and Q_10_	^65^	^8,65^	^23^

The comparative parameters are highlighted in bold.

**Fig. 7:**
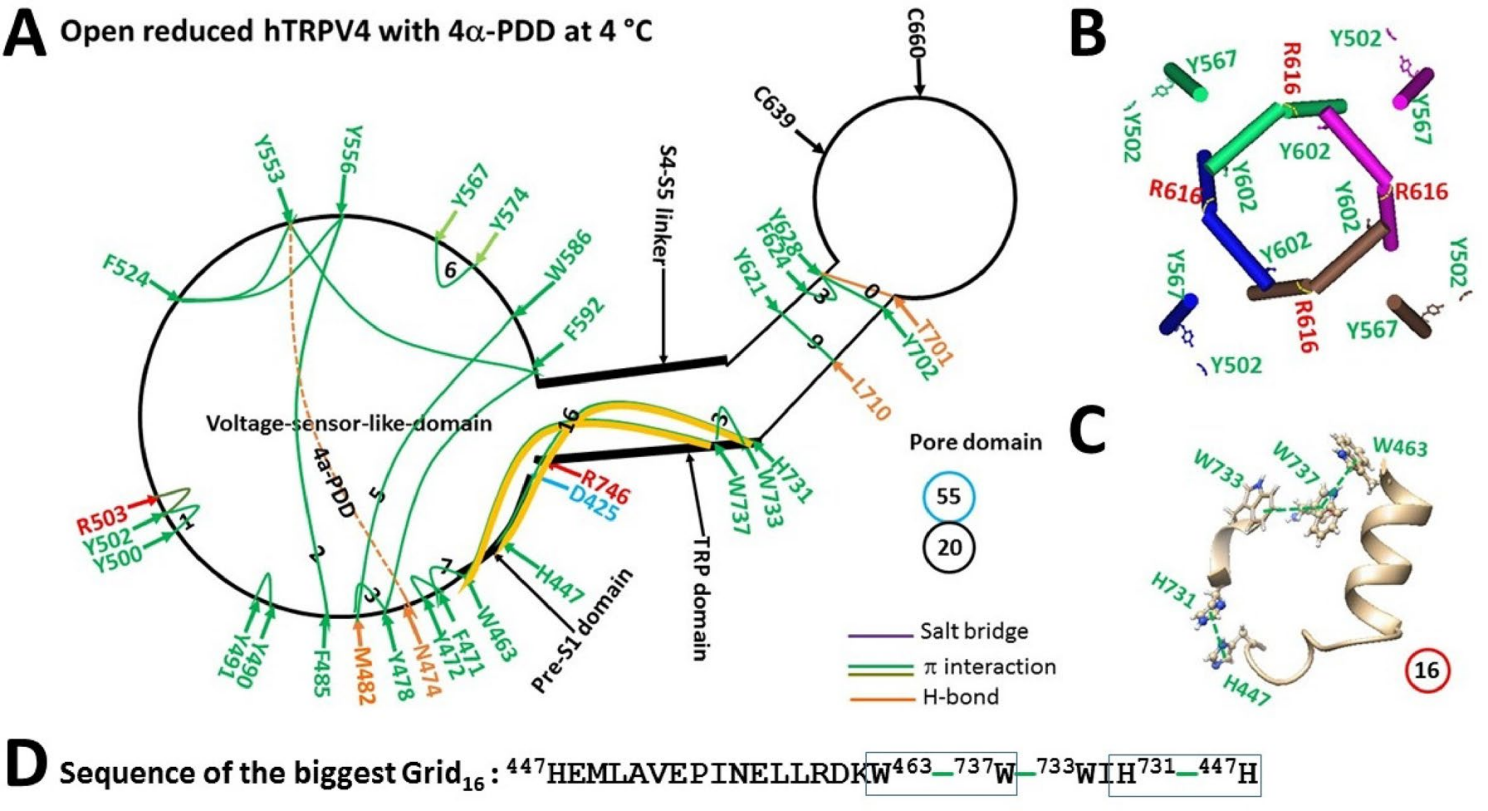
Thermoring structural rearrangement of hTRPV4 upon replacing GSK279 with 4α-PDD at low temperature. (**A**) The grid-like noncovalently interacting mesh network along the minimal gating pathway of a single subunit of reduced and open hTRPV4 with 4α-PDD bound at 4 °C (PDB: 8FCA). The pore domain, the S4-S5 linker, the TRP domain, and the pre-S1 domain are indicated by black arrows except the VSLD. Salt bridges, π interactions, and H-bonds between paired amino acid side chains along the ligand-dependent minimal gating pathway from D425 to K746 are marked in purple, green, and orange, respectively. The specific constrained grid sizes needed to control the least-stable noncovalent interactions in the grids are labeled with black numbers. The identified weakest H447-H731/W463-W737 π interactions in the biggest Grid_16_ are highlighted. The total grid sizes and the total grid size-controlled noncovalent interactions along the minimal gating pathway are shown in the cyan and black circles, respectively. (**B**) Disrupting the weakest Y502-Y567 bridges disconnected the Y602-R616’ swapping interactions near the lower gate for channel opening. (**C**) The structure of the biggest Grid_16_ with a 16-residue size to control the weakest H447-H731/W463-W737 π interactions at the pre-S1/TRP interface. (**D**) The sequence of the biggest Grid_16_ to control the weakest H447-H731/W463-W737 π interactions highlighted in the blue box.

Given that the activation of hTRPV4 by heat or the phorbol ester derivative 4α-phorbol-12,13-didecanoate (4α-PDD) is identical^65^, it is reasonable to observe that disrupting the weakest Y502/P498-Y567 bridge and then the swapping Y602-R616’ bridges promoted channel opening of hTRPV4 with RhoA/4a-PDD bound (PDB: 8FCA) (Fig. 7A–B), along with a calculated structural thermosensitivity (Ω_10_) of 20.0 similar to the experimental functionl thermosensitivity (Q_10_) of 19.1 (Table 2). Thus, either the Y502-Y567 or P498-Y567 bridge at the external interface between S1-S2 and S3-S4 linkers was further confirmed as the primary thermal sensor or trigger.

It is worth noting that the biggest Grid_16_ in 8FCA was found to regulate the H447-H731 and W463-W737 π interactions. When these two π interactions were energetically equivalent to 3.75 basic H-bonds, the calculated T_m,th_ was about 59.5 °C (Table 2), higher than the 43 °C at which the heat-evoked current is still unsaturated^65^. In this regard, 8FCA could act as a thermo-evoked open state. However, when the total numbers of noncovalent interactions and grid sizes decreased from 31 and 73 in 8FC7 to 20 and 55 in 8FCA, respectively (Figs. 6A & 7A, Tables S5 & S6), the systematic thermal instability (T_i_) also increased from 2.35 to 2.75 (Table 2). suggesting that this open state was also unstable.

### Identification of thermal sensors in hTRPV2

A recent investigation identified the weakest R367-D467 salt bridge at the internal pre-S1/S2-S3 linker interface and the weakest L555-Y590 π interaction in the external pore loop as the primary potential thermal starters for rTRPV2 activation above a threshold of 48–53 °C^68^. In that case, if disrupting the weakest R369-D469 and L555-Y590 bridges is suppressed, rTRPV2 should be inactivated.

**Fig. 8:**
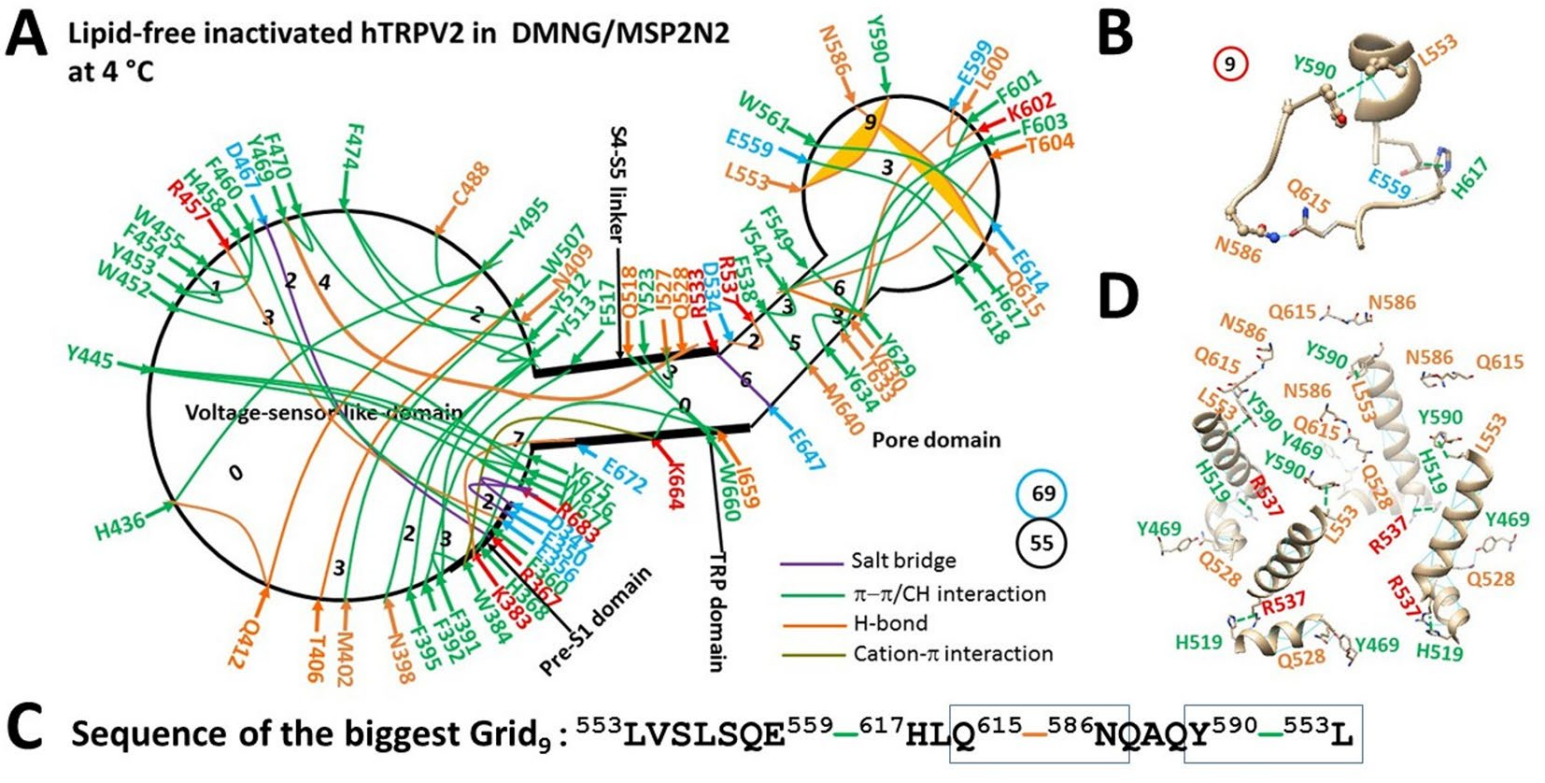
Putative thermoring structures of inactivated hTRPV2 at low temperature. (**A**) The putative grid-like noncovalently interacting mesh network along the PE-dependent minimal gating pathway of a single subunit of inactivated hTRPV2, based on a homology model of PE-free rTRPV2 in decyl maltose neopentyl glycol (DMNG) at pH 8 and 4 °C (PDB: 6U84). The pore domain, the S4-S5 linker, the TRP domain, and the pre-S1 domain are indicated by black arrows, except for the VSLD. Salt bridges, π interactions, and H-bonds between paired amino acid side chains along the PE-dependent minimal gating pathway from E347 to R683 are denoted in purple, green, and orange, respectively. The specific constrained grid sizes necessary to regulate the least-stable noncovalent interactions in the grids are indicated with black numbers. The identified weakest L553-Y590 and N586-Q615 bridges in the biggest Grid_9_ are emphasized in orange. The total grid sizes and the total grid size-controlled noncovalent interactions along the PE-dependent minimal gating pathway are displayed in cyan and black circles, respectively. (**B**) The structure of the biggest Grid_9_ with a 9-residue size to regulate the weakest L553-Y590 and N586-Q615 bridges in the pore domain. (**C**) The sequences of the biggest Grid_9_ to control the weakest L553-Y590 and N586-Q615 bridges highlighted in the blue boxes. (**D**) Allosteric gating coupling between the weakest intrsubunit L553-Y590/N586-Q615 bridges and the H519-R537’ swapping interactions near the lower gate in the inactivated state.

According to this hypothesis, when Y471 (Y469 in hTRPV2, equivalent to well-known Y511 in hTRPV1) in the S2-S3 linker H-bonded with Q530 (Q628 in hTRPV2, equivalent to famous E570 in hTRPV1) in the S4-S5 linker of phosphatidylethanolamine (PE)-free closed rTRPV2 (PDB: 6U84) via their side chains^69^, the weakest R369-D469 and L555-Y590 bridges were locked in smaller grids. Based on this homology model, the corresponding R367-D467 salt bridge in hTRPV2 was controlled by a smaller Grid_2_ via a thermoring from D467 to Y469, Q528, I527, Y523, Q518, W660, I659, H368, R367, and back to D467 (Fig. 8A, Table S7). Meanwhile, the corresponding L553-Y590 π interaction was governed by the new biggest Grid_9_ via a thermoring from L553 to E559, H617, Q615, N586, Y590, and back to L553 (Fig. 8B–C). Along with 2.5 equivalent basic H-bonds (2.5 kcal/mol) to seal the new weakest L553-Y590 and N586-Q615 bridges, the calculated T_m,th_ was about 61 °C (Table 2).

Therefore, when C488, N400, and C349 in hTRPV2 (R490, Y400 and W351 in rTRPV2, respectively) disrupt the π interactions with H436, F574 and R683 (H438, G476 and R684 in rTRPV2), respectively (Fig. 6A)^68^, the induced Y469-Q528 and N586-Q615 H-bonds may desensitize hTRPV2 possibly by increasing the activation threshold up to 61 °C, along with the swapping H519-R537’ π interctions near the lower gate^23,61,70^. In agreement with this notion, oxidation of nearby M526 and M607 by the oxidant chloramine-T or transferring the pre-S1 domain of rTRPV1 to hTRPV2 allows channel opening by heat below 61 °C^23,71^, possibly by disrupting those two inihitive H-bonds. In that case, the weakest L553-Y590 and N586-Q615 bridges may act as primary thermal sensors for a higher activation threshold in hTRPV2 above 61 °C. Notably, before oxidation, when the total noncovalent interactions and grid sizes along the lipid-dependent minimal gating pathway from D347 to R683 were 55 and 69, respectively (Fig. 8A, Table S7), the systematic thermal instability (T_i_) was calcualted as 1.25 (Table 2). Therefore, inactivated hTRPV2 may be very stable.

## Discussion

Thermosensitive TRPV1-4 channels have specific thresholds and high temperature sensitivity (Q_10_) for heat activation. While the parameters of TRPV1 and TRPV3 can be explained by a change in their thermoring structures along the PI/PC-dependent minimal gating pathway^48,49^, they have not been examined for cold activation. According to the heat capacity mechanism that extensively applies to the protein, heat-responsive TRPV1-4 channels should also be activated by cold. When both cold and heat activations have the same starting point, their Q_10_ values should be comparable^55^.

To test this hypothesis, the study first identified the thermal sensors or starters of rTRPV1-Δ(603-626) and hTRPV3 with matched thresholds for heat activation. Then, this study investigated their cold sensitivities from the same and different starting points. The results showed that the cold-evoked open state was structurally different from the heat-evoked state, even when staring from the same thermal sensor. Additionally, cold and heat activations from the same and different staring points resulted in shared and unshared temperature coefficients or sensitivities. Therefore, once the heat capacity mechanism was applied, although disrupting the common swapping π bridges near the lower gate was required for the final channel opening or activation in any case, this study confirmed that heat activation of TRPV1-4 actually began with the heat unfolding of the identified weakest noncovalent link along the defined ligand/lipid-dependent minimal gating pathway. These unique thermo-gated activation pathways have broad implications for understanding temperature-dependent gating mechanisms of thermosensitive ion channels.

### Distinct cold- and heat-evoked open states even when starting from the same thermal sensor

Removal of PI from the active vanilloid site of rTRPV1 with or without the pore turret at low and high temperatures resulted in different open states. Firstly, the stimulatory R557-E570 H-bond at the S4-S5 linker/VSLD interface (Fig. 1A), necessary for the optimal heat-evoked activity temperature of 56 °C^23,33,40,43,48,72,73^, was absent in the cold-evoked open state (Fig. 2A). Secondly, the highly conserved noncovalent interactions D576-T685 and F580-L678 in the pore domain, crucial for the maximal heat-evoked activity temperature of 61 °C^39,48,72–74^, also disappeared in the cold-evoked open state (Fig. 3A). Thirdly, in addition to the common smallest Grid_0_ consisting of Y441, F516, Y554 and Y555^48,49,72,75^, two more smallest thermorings via Y463-Y530-F543-Y463 and Y584-T641-F640-Y666-Y584 bridges appeared in the cold-evoked open state compared to the heat-evoked open state (Fig. 3A). Therefore, while the heat-evoked open state exhibited the higher melting threshold (T_m,th_) of 56 °C, the cold-evoked open state had the biggest Grid_9_ to control the least-stable F522-F543 bridge for the lower T_m,th_ of 41 °C (Table 1).

### Mirrored thermosensitivity between cold and heat activations from the same starter can be used to define or confirm the primary thermosensors

A recent study indicated that the least-stable Y401-R499 cation-π interaction in the biggest Grid_13_ along the PI-dependent minimal gating pathway from D388 to K710 is located at the internal pre-S1/VSLD interface (Fig. 1A). This interaction has a matched threshold (T_m,th_) of 43 °C for the heat activation of the full-length rTRPV1^1,23,43,48,73^. This study also confirmed that the same noncovalent bridge at the internal pre-S1/VSLD interface, but along with the H410-I696 π interaction at the internal pre-S1/TRP interface, was responsible for the matched threshold of 43 °C for the heat activation of rTRPV1 without the unstructured pore turret (603-626) (Table 1)^63^. The heat-induced unfolding of this weakest Y401-R499 bridge has been confirmed by the cryo-EM structure of open rTRPV1 above 43 °C (Fig. 1A)^40^. However, it is unclear if the heat activation of rTRPV1 primarily stems from the heat-induced unfolding of this Y401-R499 bridge at the specific threshold. Since the cold activation of rTRPV1-Δ(603-626) was directly initiated by the removal of PI from the active vanilloid site and the concurrent disruption of the least-stable Y401-R499 and H410-I696 bridges along the same PI-dependent minimal gating pathway from D388 to K710 (Figs. 2–3), the comparable cold and heat sensitivity of 21 demonstrated that both cold and heat activations did start from the unfolding of the weakest Y401-R499 and H410-I696 bridges at the specific temperature threshold (Table 1).

In contrast, similar to the least-stable K614-N647 H-bond in the biggest Grid_11_ in the pore domain of reduced mTRPV3 (Fig. 1B)^49^, the weakest E610-K649 H-bond in the biggest Grid_14_ of reduced hTRPV3 also had a matching threshold of 52 °C for heat activation (Fig. 4, Table 1)^32^. However, when the K169A mutation-induced cold activation at 4 °C did not disrupt this weakest E610-K649 H-bond or salt bridge, the calculated structural thermosensitivity (Ω_10,cold_) of 0.39 was significantly lower than the measured functional heat sensitivity (Q_10, heat_) of 22.2 (Table 1)^32^. This lower cold sensitivity was consistent with the slow channel dilation of the K169A mutant at the S2-S3 linker from 42 °C to 25 °C when compared with the stimulus of carvacrol or 2-aminoethoxydiphenylborane (2-APB)^76,77^. In this regard, according to the heat capacity mechanism^55^, the primary heat activation of hTRPV3 above 50 °C was initiated by disrupting the weakest E610-K649 bridge at the external interface between two pore turrets, rather than the swapping K169-E751’ salt bridge (Fig. 4A–C)^32,49^.

Given that *Xenopus* oocytes expressing frog TRPV3 (fTRPV3) exhibit a higher Q_10_ of 71 upon heat activation above 50 °C compared to cold activation below 20 °C^78,79^, it is possible that heat activation may start with the unfolding of the weakest E589-K624 (E610-K649 in hTRPV3) bridge at the interface between two pore turrets (Fig. 4A). However, a potential electronic repulsion between K149 (K169 in hTRPV3) and K726 (E751 in hTRPV3) may induce cold activation. In addition, since redox-dependent cold and heat activations of hTRPA1 at 22 °C are not strictly symmetric, it is necessary to further examine if these two events have the same or different triggers^80^. Finally, although phosphatidylinositol-4,5-biphosphate (PIP_2_) and chemical agents such as cryosim-3 (C3) and allyl isothiocyanate (AITC) can open mouse TRPM8 (mTRPM8) with the weakest F874-Y908 π bridge in the pore domain to regulate the cold threshold of 20 °C^42,50^, long-chain intracellular lysophospholipids can activate mTRPM8 even at normal body temperature^81,82^. Further study is necessary to examine if the least-stable bridge in the pore domain serves as a thermal sensor to adjust the activation of mTRPM8 by lysophospholipids with a heat sensitivity to mirror the cold sensitivity in the presence of PIP_2_.

### Implications of thermo-gated activation pathways of TRPV1-4 biothermometers

Timing is crucial for understanding the activation pathways of thermo-gated TRP channels. However, recent steady-state cryo-EM structures of TRPV1, TRPV3 or TRPM8 at low and high temperatures lack the time-resolved conformational landscapes^40–42^. On the other hand, the highly conserved swapping interactions (Y565-R579’ in TRPV1, Y575-K589’ in TRPV3, Y602-R616’ in TRPV4 or H519-R537’ in TRPV2) near the lower gate must be disrupted for the final thermo-gated activation of TRPV1-4 channels (Figs. 1, 2D, 3B, 4D, 5A, 6D, 7B, 8D)^39–41,56,57,59–62,66,68,69^. Thus, various pathways to disrupt these conserved swapping interactions result in distinct temperature thresholds for activation.

For reduced TRPV1 or oxidized TRPV3, unfolding the weakest bridge at the internal interface between the S2-S3 linker and the pre-S1 domain (Y401-R499 in rTRPV1, E406-K504 in hTRPV1 or R416-D519 in oxidized mTRPV3) primes PI/PC release from the active vanilloid site for channel activation above 41 °C^40,41,48,49^. However, for reduced TRPV3, unfolding the weakest bridge at the external interface of two pore turrets (K614-N647 in mTRPV3 or E610-K649 in hTRPV3) can also release PC for channel activation above 50 °C (Fig. 1) ^32,39,49^. Further, for rTRPV2, unfolding not only the weakest R369-D469 bridge at the internal pre-S1/VSLD interface but also the least-stable L555-Y590 bridge in the external pore loop is required to release PE from the VSLD for channel activation above 48–53 °C^68^. Finally, unfolding the weakest Y502/P498-Y567 bridge at the external interface between S1-S2 and S3-S4 linkers may be required to release the inhibitor from the VSLD for hTRPV4 activation above 26/34 °C (Figs. 6, 7, Table 2)^8,9,65,67^.

In support of the heat-evoked activation pathways proposed above, the T406D mutation in rTRPV1 (equivalent to T407 in hTRPV1) slows down the heat activation process but phosphorylation of the nearby S502 by protein kinase C sensitizes TRPV1^83–85^. Moreover, the insertion of valine at position 412 in mTRPV3 (equivalent to 405 in rTRPV1 or 406 in hTRPV1) allows the weakest R416-D519 salt bridge to lower the initial activation threshold from 52 °C to 42 °C^30,49^. Similarly, swapping the pre-S1 domain from rTRPV1 into rTRPV2, hTRPV2, or mTRPV4 alters their heat sensation to a different extent^23^. Specifically, replacing the segment ^365^KD^366^ of rTRPV2 with the equivalent ^405^ET^406^ of rTRPV1 reduces the activation threshold T_th_ from 52 °C to 46 °C^36^. However, the mutation N643S, I644S, N647Y, Y661C, or L657I in the pore domain can desensitize TRPV3^21^. Since Y555A rather than Y555F (Y556 in hTRPV4) suppresses the activation of murine TRPV4 by heat and 4α-PDD rather than cell swelling^86^, the conserved F485-Y556-F524 bridges may serve as an indispensable anchor for the heat response of hTRPV4 (Figs. 6A, 7A). Further, given that disrupting Y110 phosphorylation or the binding of PACSIN 3 to nearby P142 and P143 (P143 and P144 in hTRPV4) in the ARD suppresses activation of murine TRPV4 by heat and cell swelling rather than 4α-PDD^87–89^, the structural perturbation in the ARD may allosterically affect the F485-Y556-F524 anchor bridges (Figs. 6A, 7A). In the case of hTRPV2, if unfolding the R367-D467/L553-Y590 bridges is prohibited by the Y469-Q528 and N586-Q615 H-bonds (Fig. 8), the channel may become unresponsive to heat below 61 °C until the nearby M607 and M526 are oxidized^23,69–71^.

On the other hand, directly removing PI/PC/PE from TRPV1/TRPV3/TRPV2 or replacing the inhibitor with an agonist in TRPV4 at low temperature can also disrupt the highly-conserved intersubunit interactions near the lower gate for cold activation (Figs. 2, 3, 4, 5, 6, 7)^68^. Therefore, different unfolding pathways from the specific weakest tertiary noncovalent interactions to the specific quaternary noncovalent bridges prime temperature-dependent activation of thermosensitive TRPV1-4 channels and define their distinct biothermometers with specific thresholds. These findings have broad implications for understanding other thermo-gated ion channels including TRPM2-5/8, TRPA1 and TRPC5.

Overall, although the ARD, pre-S1 domain, S1-S4 or VSLD domain, pore domain, TRP helix and CTD have been suggested as the primary thermal sensors or triggers for specific activation thresholds of the full-length TRPV1-4 channels^17–38^, the thermoring analyses based on the heat capacity mechanism in this study demonstrated that their primary thermal sensors could be located at the internal interface between the pre-S1 domain and the S2-S3 linker, the external interface between S1-S2 and S3-S4 linkers, or the external interface between two pore turrets. Further mutations and electrophysiological measurements are necessary to examine these primary thermal sensors.

## Conclusions

Most heat-responsive TRPV1-4 channels have a higher activation threshold for detecting environmental noxious heat stimuli. However, the primary modules for these specific thresholds have not been precisely defined due to a global cooperative conformational change across the entire protein during channel opening. This study demonstrates that an alternative cold activation pathway with a mirrored heat coefficient could be used to precisely define the primary modules for the specific thresholds if the same heat capacity mechanism for cold and heat unfolding transitions is involved. Therefore, even though different starting points trigger distinct cold or heat unfolding pathways allosterically, the symmetric temperature coefficient can always be used to precisely define their common staring point as the primary module for the specific threshold.

## Materials and methods

### Cryo-EM structures used

The cryo-EM 3D structures of the reduced hTRPV3 channel with MSP2N2 in the closed state at 4 °C (PDB: 6UW4, model resolution = 3.20 Å) and the reduced hTRPV3-K169A channel with MSP2N2 in the open state at 4 °C (PDB: 6UW6, model resolution = 3.70 Å) were analyzed to study K169A-induced cold sensitivity^60^. As controls, the cryo-EM 3D structures of the reduced rTRPV1-Δ(604-626) channel with MSP2N2 in the closed state at 4 °C (PDB: 5IRZ, model resolution = 3.28 Å) and in the open state at 25 °C (PDB: 8U3L, model resolution = 3.70 Å) were studied to analyze cold sensitivity upon PI removal^56,57^. In addition, the full-length cryo-EM structures of closed hTRPV4-RhoA with GSK2798745 bound (PDB: 8FC7, model resolution = 3.3 Å) and presumably open hTRPV4-RhoA with 4α-PDD bound at 4 °C (PDB: 8FCA, model resolution = 3.41 Å) were used to identify the primary potential thermal sensor in hTRPV4^66^. Finally, the full-length cryo-EM structure of apo rTRPV2 in nanodiscs, state 1 (PDB: 6U84, model resolution = 3.7 Å) was used as a homology model of inactivated hTRPV2 to define its potential thermal sensor^69^.

### Defining the minimal gating pathway

Recent studies have shown that the polypeptide chain, spanning from D388 or D396 in the pre-S1 domain to K710 or K705 in the TRP domain of TRPV1 or TRPV3, is sufficient to create a network of tertiary noncovalent interactions. Once these interactions are constrained as thermorings, they can account for the measured temperature thresholds and sensitivities. Therefore, that chain has been defined as the minimal gating pathway^48,49,51^. In this computational study, a similar minimal gating pathway from D425 in the pre-S1 domain to R746 in the TRP domain of hTRPV4 was found to explain the experimental thresholds and thermosensitivity as well. However, for hTRPV2 inactivation below 61 °C, the minimal gating pathway from D342 to R683 was necessary.

### Filtering tertiary noncovalent interactions

The stereo-selective and regio-selective inter-domain diagonal and intra-domain lateral noncovalent interactions along the above defined minimal gating pathway of TRPV1-4 were primarily analyzed using UCSF Chimera. The interactions were then filtered by the same strict and consistent standard as previously used and confirmed^45–54^. The examined noncovalent interactions included salt bridges, lone pair/CH/cation- π interactions and H-bonds between paired amino acid side chains. Specific cutoff distances and interaction angles for the different noncovalent interactions can be found in the online Supporting Information (Table S1–S7). Notably, momentary fluctuation-induced perturbations in tertiary noncovalent interactions during protein dynamics were not considered.

### Mapping the energy landscape of thermoring structures of TRPV1-4 using the grid thermodynamic model

The same protocol that was previously described and validated was used in this study to map the systematic fluidic grid-like noncovalent interaction mesh network^45–54^. In this network, a topological grid consisted of several nodes representing amino acids, with linked nodes representing noncovalent interactions along the single polypeptide chain. Graph theory and the Floyd–Warshall algorithm were used to determine the grid size as the shortest round path length to control the least-thermostable noncovalent interaction within the grid^90^. The grid size also represented the minimal number of side chains of free or silent amino acids that did not participate in any tertiary noncovalent interaction within the grid. Uncommon grid sizes were denoted in black numbers on the network map alongside the Grid_s_ with an s-residue size.

Once the tertiary noncovalent interactions networks were constrained as thermorings with the minimum energy required to stabilize the interactions, the biggest thermoring could be identified to trace the weakest noncovalent interaction along the defined minimal gating pathway. Meanwhile, the total noncovalent interactions (*N*) and total grid sizes (*S*) along the PI, PC, ligand or PE-dependent minimal gating pathway of rTRPV1-Δ(604-626), hTRPV3/K159A, hTRPV4 or hTRPV2 were calculated and displayed in black and cyan circles, respectively, next to the mesh network map for the calculation of systematic thermal instability (T_i_).

### Calculating the melting temperature threshold (T_m,th_)

The melting temperature threshold (T_m,th_) for the heat-induced unfolding of a specific grid was determined using an empirical equation and coefficients calibrated based on temperature-dependent structural data from various proteins, including class I and II fructose aldolases, TRPV1, TRPV3, and TRPM8^45–54^:

1 $${\mathrm{T}}_{{{\mathrm{m}},{\mathrm{th}}}} (^\circ {\mathrm{C}}) = {34} + \left( {{\mathrm{n}} - {2}} \right) \times {1}0 + \left( {{2}0{-}{\mathrm{s}}} \right) \times {2}$$

where, n represents the total number of basic H-bonds (each approximately 1 kcal/mol) that are calculated to be roughly equivalent in stability to the least-stable noncovalent interaction controlled by the specific grid^91^. The variable s denotes the grid size used to regulate the least-stable noncovalent interaction within the grid. Thus, the grid’s heat capacity will increase with a decrease in the grid size or an increase in the number of equivalent basic H-bonds.

### Evaluating the grid-based systemic thermal instability (T_i_)

The same empirical equation used in previous studies on temperature-dependent structures was utilized to calculate the systematic thermal instability (T_i_) along the given polypeptide chain^45–54^:

2 $${\mathrm{T}}_{{\mathrm{i}}} = {S \mathord{\left/ {\vphantom {S N}} \right. \kern-0pt} N}$$

where, *S* and *N* are the total grid sizes and non-covalent interactions along a specific polypeptide chain. This calculation allows for evaluating the protein’s compact conformational entropy or flexibility.

### Evaluating the systematic temperature sensitivity

A gating transition of the thermosensitive TRPV1 or TRPV3 channel is always accompanied by a change in the conformational energy density along the lipid-dependent minimal gating pathway^48,49^. Accordingly, for enthalpy-driven activation of TRPV1 or TRPV3 from a closed state within 10 °C as a result of the broken weakest bridge, if the chemical potential of a grid is theoretically defined as the maximal potential for equivalent residues in the grid to form the tightest β-hairpin with the smallest loop via noncovalent interactions^92^, the grid-based structural thermo-sensitivity (Ω_10_) of a single ion channel for cold activation could be defined and calculated using the following equations as examined previously^48,49^.

3 $$\Omega_{{{1}0}} = \left[ {\left( {{\mathrm{S}}_{{\mathrm{c}}} {-}{\mathrm{S}}_{{\mathrm{o}}} } \right){{\mathrm{E}} \mathord{\left/ {\vphantom {{\mathrm{E}} {2}}} \right. \kern-0pt} {2}}} \right]^{{({{{\mathrm{Hc}}} \mathord{\left/ {\vphantom {{{\mathrm{Hc}}} {{\mathrm{Ho}}}}} \right. \kern-0pt} {{\mathrm{Ho}}}})}} = \left[ {\left( {{\mathrm{S}}_{{\mathrm{c}}} {-}{\mathrm{S}}_{{\mathrm{o}}} } \right){{\mathrm{E}} \mathord{\left/ {\vphantom {{\mathrm{E}} {2}}} \right. \kern-0pt} {2}}} \right]^{{[({{{\mathrm{ENc}}} \mathord{\left/ {\vphantom {{{\mathrm{ENc}}} {({\mathrm{ENo}})}}} \right. \kern-0pt} {({\mathrm{ENo}})}}]}} = \left[ {\left( {{\mathrm{S}}_{{\mathrm{c}}} {-}{\mathrm{S}}_{{\mathrm{o}}} } \right){{\mathrm{E}} \mathord{\left/ {\vphantom {{\mathrm{E}} {2}}} \right. \kern-0pt} {2}}} \right]^{{({{{\mathrm{Nc}}} \mathord{\left/ {\vphantom {{{\mathrm{Nc}}} {{\mathrm{No}}}}} \right. \kern-0pt} {{\mathrm{No}}}})}}$$

where, along the same defined PI, PC, ligand or PE-dependent minimal gating pathway of one subunit, N_c_ and N_o_ represent the total noncovalent interactions, H_c_ and H_o_ denote the total enthalpy included in them, and S_c_ and S_o_ indicate the total grid sizes in the closed and open states, respectively. The energy intensity of a noncovalent interaction is denoted by E and is typically 1 kcal/mol. Thus, Ω_10_ factually reflects a thermo-evoked change in the total chemical potential of grids upon a thermo-evoked change in the total enthalpy included in the noncovalent interactions apparently from a closed state to an open state along the same defined PI, PC, ligand or PE-dependent minimal gating pathway of one subunit.

For a convenient comparison, the functional thermo-sensitivity (Q_10_) of a single ion channel for heat activation can be calculated using the following equation:

4 $${\mathrm{Q}}_{{{1}0}} = \left( {{{{\mathrm{X}}_{{2}} } \mathord{\left/ {\vphantom {{{\mathrm{X}}_{{2}} } {{\mathrm{X}}_{{1}} }}} \right. \kern-0pt} {{\mathrm{X}}_{{1}} }}} \right)^{{{{{1}0} \mathord{\left/ {\vphantom {{{1}0} {({\mathrm{T2}} - {\mathrm{T1}})}}} \right. \kern-0pt} {({\mathrm{T2}} - {\mathrm{T1}})}}}}$$

where, X_1_ and X_2_ are the relative channel activity obtained at temperatures T1 and T2 (measured in Kelvin), respectively.

## Supplementary Information

Below is the link to the electronic supplementary material.

Supplementary Material 1

## Data Availability

All data generated or analysed during this study are included in this published article and Supporting Information.

## Abbreviations

AITC: Allyl isothiocyanate
ARD: Ankyrin repeat domain
C3: Cryosim-3
cryo-EM: Cryogenic electron microscopy
CTD: C-terminal domain
DMNG: Decyl maltose neopentyl glycol
DSC: Differential scanning calorimetry
GSK279: GSK2798745
Ω_10_: Structural thermosensitivity
Q_10_: Functional thermosensitivity
4α-PDD: 4α-Phorbol-12,13-didecanoate
LMNG: Lauryl maltose neopentyl glycol
MSP2N2: Membrane scaffold protein 2N2
PI: Phosphatidylinositol
PIP_2_: Phosphatidylinositol-4,5-biphosphate
PC: Phosphatidylcholine
PE: Phosphatidylethanolamine
T_i_: Systematic thermal instability
T_m,th_: Melting temperature threshold
TRP: Transient receptor potential
TRPV: TRP vanilloid
TRPVi: (I = 1, 2, 3, 4)
TRPM2-5, 8: TRP melastatin 2–5, 8
TRPA1: TRP ankyrin 1
TRPC5: TRP canonical 5,
fTRPV3: Frog TRPV3
rTRPV1: Rat TRPV1
rTRPV2: Rat TRPV2
rTRPV4: Rat TRPV4
hTRPV1: Human TRPV1
hTRPV2: Human TRPV2
hTRPV3: Human TRPV3
hTRPV4: Human TRPV4
mTRPV3: Mouse TRPV3
mTRPV4: Mouse TRPV4
mTRPM8: Mouse TRPM8
VSLD: Voltage sensor-like domain
2-APB: 2-Aminoethoxydiphenylborane
