# Supplementary material for: Pathway-dependent cold activation of heat-responsive TRPV channels
Source: Sci Rep. 2025 Dec 1;15:45041. doi: 10.1038/s41598-025-29524-y (PMC12749286; doi:10.1038/s41598-025-29524-y)
Supplement: Supplementary file 1 — Supplementary Material 1 [file 41598_2025_29524_MOESM1_ESM.pdf]

Supporting Information for

Pathway-dependent cold activation of heat-responsive TRPV channels

Guangyu Wang 1, 2*

[1]Department of Physiology and Membrane Biology, University of California School of Medicine, Davis, CA, USA

[2]Department of Drug Research and Development, Institute of Biophysical Medico-chemistry, Reno, NV, USA

* Correspondence: gary.wang10@gmail.com

This Supporting Information includes:

Tables S1, S2, S3, S4, S5, S6, S7 and Figure S1.

Table S1 Tertiary noncovalent interactions along the PI-dependent minimal gating pathway from I387 to K710 in each subunit of closed PI-bound rTRPV1-Δ(604-626) in MSP2N2 at 4 °C (PDB ID, 5IRZ)

Noncovalent interaction	Cut-off distance	Linked residues
Salt bridge	3.2-4 Å	D471-R474, D576-R579
H-bond	<3.9 Å	I387/D388-S394, S402-R409, **K425-E709**, Y441-Q519, T449-W549, S512-PI-R557, Q519-N551, R557-PI-E570, **Y584-T641**/Y666, S629-S632
π–π interaction	2.65–6.5 Å	**F434/F438**/Y441-**Y555**, **Y441-Y444/F488/F516, Y444-F488, Y453-Y454, Y463-Y530**, **Y487-F488, F488-F516**, **F489-**F490/**F517**, **F516-Y554, F522-F543, Y530-F531, Y530-Y537, Y554-Y555, F591-Y666, Y627-F649, F640-Y666**, **F649/Y653-F659**
cation–π interaction	<6.0 Å	Y401-R499, **W426-R701**
CH_3/CH–π interaction	2.65-3.01 Å	H410-I696, **Y444-F448, M445-W549,** P456-Y463, F489-L524, **Y495-E513**, F580-L678, F580-F584, **F589-T593, E636-F649,** E651-Y653
Lone pair–π interaction	3-3.7 Å	Y666-T670

Note: Bold interactions were conserved in both closed and open states of rTRPV1-Δ(604-626).

Table S2 Tertiary noncovalent interactions along the PI-dependent gating pathway from I387 to K710 in each subunit of open PI-free rTRPV1-Δ(604-626) in MSP2N2 at 25 °C (PDB ID, 8U3L)

**Noncovalent interaction**	**Cut-off distance**	**Linked residues**
Salt bridge	3.2-4 Å	R534-E536, E636-K639
H-bond	<3.9 Å	S403-T406, E416-R420, **K425-E709,** Y463-Y537, Q498-R499, S502-S505, S512-E513, **Y584-T641**
π–π interaction	2.65–6.5 Å	**F434-W426/Y555,** F438-Y555, **Y441-Y444/ F488/F516/Y555, Y444-F488, Y453-Y454, Y463-Y530, Y487-F488-F516, F489-F517,** Y495-F496, **F516-Y554, F522-F543, Y530-F531/Y537, Y554-Y555,** Y584-Y666, **F591-Y666, Y627-F649, F640-Y666, F649/Y653-F659**
cation–π interaction	<6.0 Å	**W426-R701**
CH_3/CH–π interaction	2.65-3.01 Å	**Y444-F448, M445-W549, Y495-E513,** Q560-W697, **F589-T593, E636-F649,** D654-F655
Lone pair–π interaction	3-3.7 Å	

Note: Bold interactions were conserved in both closed and open states of rTRPV1-Δ(604-626).

Table S3. Tertiary noncovalent interactions along the PC-dependent minimal gating pathway from D396 to K705 in each subunit of the closed reduced hTRPV3 channel at 4 °C (PDB ID, 6UW4).

Noncovalent interaction	Cut-off distance	Linked residues
Salt bridge	3.2-4 Å	E405-K705, E418-R690, K500-E501, PC-R567, **E689-R693**
H-bond	<3.9 Å donor-H-acceptor <60°	**D396-K432-E704**, Y409-E702, R416-D519, Y448-**Q529-Y451**, **T456-W559**, E501-H523,T566-S576, **D586-T680**, **Y594-Y661**, **E610-K649**, E631-K634
π–π interaction	2.65–6.5 Å	H426-H430, W433-**F441-Y565**, **F449-F445-Y565**, **F447-W493-F489**-Y451, **Y448-Y451**, **Y448-F526**, **Y448-Y565**, **F449-W559**, **Y451-W493**, Y460-Y461, **W521-F522-Y564, F526-Y564**, **Y540-Y547,** F542-Y544, **Y564-Y565**, **F601-Y661**, **Y622-F654**
cation–π interaction	<6.0 Å	**W433-R696**
CH_3/CH–π interaction	2.65-3.01 Å	**W433-K438, N452-W559**, W521-PC, F527-V531, **Q570-W692**, **F590-L673**, F597-L664, L632-Y661, F633-I637
Lone pair–π interaction	3-3.7 Å	F656-T660, **T665-Y661**

Note: Bold interactions were conserved in both closed hTRPV3 and open hTRPV3/K169A.

Table S4. Tertiary noncovalent interactions along the PC-dependent minimal gating pathway from D396 to K705 in each subunit of the open reduced hTRPV3-K169A channel at 4 °C (PDB ID, 6UW6).

**Noncovalent interaction**	**Cut-off distance**	**Linked residues**
Salt bridge	3.2-4 Å	**E610-K649. E689-R693**, R698-E702
H-bond	<3.9 Å donor-H-acceptor <60°	**D396-K432-E704,** T397-E704, N412-D512, **Q529-Y451**, **T456-W559**, D519-R567, **D586-T680**, **Y594-Y661**, Y594-T636-Y661
π–π interaction	2.65–6.5 Å	**F441-Y565**, **F449-F445-Y565**, **F447-W493-F489**, **Y448-Y451, Y448-F526, Y448-Y565, F449-W559**, **Y451-W493**, **W521-F522-Y564**, **F526-Y564**, **Y540-Y547, Y564-Y565**, **F601-Y661**, **Y622-F654**,
cation–π interaction	<6.0 Å	**W433- R696**
CH_3/CH–π interaction	2.65-3.01 Å	**W433-K438**, **N452-W559**, **Q570-W692**, F590-Y594, **F590-L673**
Lone pair–π interaction	3-3.7 Å	**T665-Y661**

Note: Bold interactions were conserved in both closed hTRPV3 and open hTRPV3/K169A.

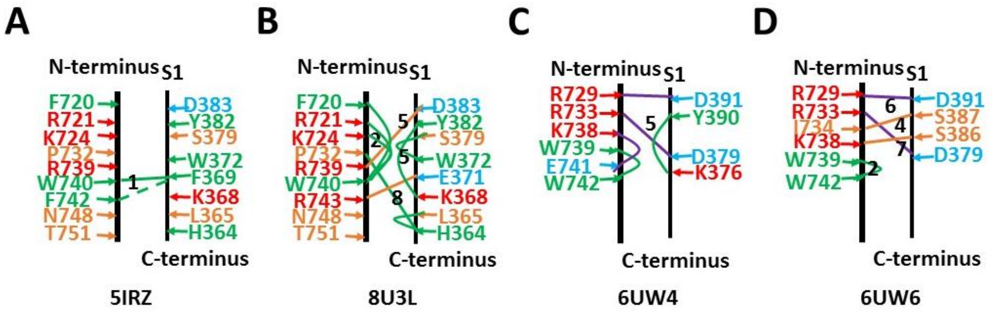

Fig.S1. **The grid-like noncovalently interacting mesh network between N- and C-terminal domains of reduced rTRPV1-Δ(604-626) or hTRPV3 beyond the PI/PC-dependent minimal gating pathway.** (**A)** The reduced and closed rTRPV1-Δ(604-626) channel in MSP2N2 at 4 °C (PDB ID, 5IRZ). (**B)** The reduced and open rTRPV1-Δ(604-626) channel in MSP2N2 at 25 °C (PDB ID, 8U3L). (**C)** The reduced and closed hTRPV3 channel in MSP2N2 at 4 °C (PDB ID, 6UW4). (**D)** The reduced and open hTRPV3-K169A channel in MSP2N2 at 4 °C (PDB ID, 6UW6). The N- and C-terminal domains are indicated in black along with S1. Salt bridges, π interactions, and H-bonds between paired amino acid side chains are denoted in purple, green, and orange, respectively. The specific grid sizes necessary to regulate the least-stable noncovalent interactions in the grids are indicated with black numbers.

Table S5. Tertiary noncovalent interactions along the ligand-dependent minimal gating pathway from D425 to R746 in each subunit of the closed reduced hTRPV4 channel with RhoA/GSK279 bound at 4 °C (PDB ID, 8FC7).

Noncovalent interaction	Cut-off distance	Linked residues
Salt bridge	3.2-4 Å	D425-K462, E435-R746, K727-E728, D743-R746
H-bond	<3.9 Å donor-H-acceptor <60°	Y439-R746, K462-E745, Y502-Y567
π–π interaction	2.65–6.5 Å	**W737-W463-F471-Y472**, F471-**F592-Y478**-F524/Y553, **F485-Y556-F524-Y553**, **Y490-Y491**, F549/Y553-Y591, **Y567-Y574**, Y591-F592, F617-Y621, **F624-Y628-Y702**, **W733-W737**
cation–π interaction	<6.0 Å	
CH_3/CH–π interaction	2.65-3.01 Å	**M482-W586**, (P498-Y567), F525-I529, K597-W733, Y624-L709, F707-L711
Lone pair–π interaction	3-3.7 Å	

Note: Bold interactions were conserved in both closed and open hTRPV4.

Table S6. Tertiary noncovalent interactions along the ligand-dependent minimal gating pathway from D425 to R746 in each subunit of the open reduced hTRPV4 channel with 4α-PDD bound at 4 °C (PDB ID, 8FCA).

Noncovalent interaction	Cut-off distance	Linked residues
Salt bridge	3.2-4 Å	
H-bond	<3.9 Å donor-H-acceptor <60°	Y628-T701
π–π interaction	2.65–6.5 Å	H447-H731, **W737-W463-F471-Y472, F592-Y478, F485-Y556**, **Y490-Y491**,Y500-Y502, **Y556-F524-Y553-**F592, **Y567-Y574**, **F624-Y628-Y702, W733-W737**
cation–π interaction	<6.0 Å	Y502-R503
CH_3/CH–π interaction	2.65-3.01 Å	Y621-L710, Y478-**M482-W586**
Lone pair–π interaction	3-3.7 Å	

Note: Bold interactions were conserved in both closed and open hTRPV4.

Table S7. Tertiary noncovalent interactions along the PE-dependent minimal gating pathway from D347 to R683 in each subunit of closed hTRPV2 homology model based on close state 1 of rTRPV2 in DMNG/MSP2N2 at pH 8 and 4 °C (PDB ID, 6U84).

Noncovalent interaction	Cut-off distance	Linked residues
Salt bridge	3.2-4 Å	D347/E350-R683, R367-D467, R533-E647
H-bond	<3.9 Å donor-H-acceptor <60°	E356-R457, K383-E672, T406-W507, Q412-H436/Y495, Y469-Q528, D534-R537, Y542-T604/Y629, L553-Y590, N586-Q615, E599-K602, L600-Y629
π–π interaction	2.65–6.5 Å	F360-F460, W384-F391-Y513, F392-F517, F395-Y513, H436-Y495, Y445-Y675/W676/W677, W452-W676, Y453-F454/F460, W455-H458, Y469-F470, F470-Y512, F474-Y513, Y512-Y513, F538-Y542, Y542-F601, F549-Y629, F603-Y629, H617-F618, W676-W677
cation–π interaction	<6.0 Å	W384-K664
CH_3/CH–π interaction	2.65-3.01 Å	H368-I659, N398-Y513, M402-W507, F474-N409, C488-Y495, Q518-W660, Y523-I527, F538-M640, F603-V630-Y634, W660-K664
Lone pair–π interaction	3-3.7 Å	E559-H617, W561-E614, Y629-T633
